# The Mediterranean Sea as a barrier to gene flow: evidence from variation in and around the F7 and F12 genomic regions

**DOI:** 10.1186/1471-2148-10-84

**Published:** 2010-03-27

**Authors:** Georgios Athanasiadis, Emili González-Pérez, Esther Esteban, Jean-Michel Dugoujon, Mark Stoneking, Pedro Moral

**Affiliations:** 1 Unitat d'Antropologia, Facultat de Biologia, Universitat de Barcelona, Barcelona, Spain; 2 CNRS and University Toulouse III Paul Sabatier, Toulouse, France; 3 Department of Evolutionary Genetics, Max Planck Institute for Evolutionary Anthropology, Leipzig, Germany

## Abstract

**Background:**

The Mediterranean has a long history of interactions among different peoples. In this study, we investigate the genetic relationships among thirteen population samples from the broader Mediterranean region together with three other groups from the Ivory Coast and Bolivia with a particular focus on the genetic structure between North Africa and South Europe. Analyses were carried out on a diverse set of neutral and functional polymorphisms located in and around the coagulation factor VII and XII genomic regions (F7 and F12).

**Results:**

Principal component analysis revealed a significant clustering of the Mediterranean samples into North African and South European groups consistent with the results from the hierarchical AMOVA, which showed a low but significant differentiation between groups from the two shores. For the same range of geographic distances, populations from each side of the Mediterranean were found to differ genetically more than populations within the same side. To further investigate this differentiation, we carried out haplotype analyses, which provided partial evidence that sub-Saharan gene flow was higher towards North Africa than South Europe.

**Conclusions:**

As there is no consensus between the two genomic regions regarding gene flow through the Sahara, it is hard to reach a solid conclusion about its role in the differentiation between the two Mediterranean shores and more data are necessary to reach a definite conclusion. However our data suggest that the Mediterranean Sea was at least partially a barrier to gene flow between the two shores.

## Background

The history of the Mediterranean involves successive population movements across the lands that surround it, both in prehistoric and historical times. In historical times, these population movements have included peoples like Greeks, Romans, Celts, Goths, Slavs, Arabs and Turks[1]. It is thus a great challenge - as the great number of relevant human population genetic studies also reveals - to investigate the extent to which this intense migratory activity has influenced the genetic composition of the present Mediterranean populations.

Regarding the Mediterranean genetic profile, a recent X chromosome SNP study showed that the region exhibits a high overall genetic homogeneity,[2] which seems to agree with an apparently weak genetic structure between South Europeans and North Africans, as revealed by an analysis of Y chromosome microsatellites[3]. This pattern may be a consequence of the Neolithic demic diffusion in this region (around 10,000 years before present) and/or a high level of gene flow in the area.

In any case, the genetically homogeneous Mediterranean landscape is sprinkled with differentiated isolates such as the Corsicans,[4] the Sardinians[5] and populations from the Balearic Islands[6]. Moreover, a Moroccan sample was found to present significant genetic differences from other Mediterranean populations in their X chromosomes[2]. This last observation has been attributed by some scholars to the potential role of the Gibraltar Strait as a genetic barrier between Northwest Africa and the Iberian Peninsula,[7] although there is no general consensus on this issue,[8,9] possibly reflecting the fact that different markers and genomic components reveal different patterns.

In this study we investigate the genetic structure of human populations in the Mediterranean, with a particular emphasis on the genetic relationships between groups from North Africa and South Europe. We paid special attention to the role of gene flow through the Sahara in the genetic differentiation between Northern Africans and Southern Europeans. To accomplish our goals, we used polymorphisms in and around the genomic regions of the F7 and F12 genes. These genes code for the coagulation factors VII and XII respectively and are involved in blood clotting. The chosen polymorphisms from the functional regions of the two genes were previously reported to be associated with susceptibility to cardiovascular disease in groups from the Mediterranean[10,11].

Some of the data used here (i.e. variation in and around the F7 gene) were published previously,[12] while new data include neutral variation around the F12 gene and the F12 46C>T functional polymorphism. This extensively studied marker is related to Factor XII plasma levels and the development of thrombosis, although the causal relationship between these two features is questionable[13].

According to our data, the Mediterranean populations are significantly clustered into South Europeans and North Africans, despite the low genetic differentiation between the two groups. Our analyses also suggest that this differentiation can be explained by the Mediterranean Sea acting a genetic barrier, which may also have affected the sub-Saharan gene flow into the Mediterranean region.

## Methods

### Samples

**Figure 1:**
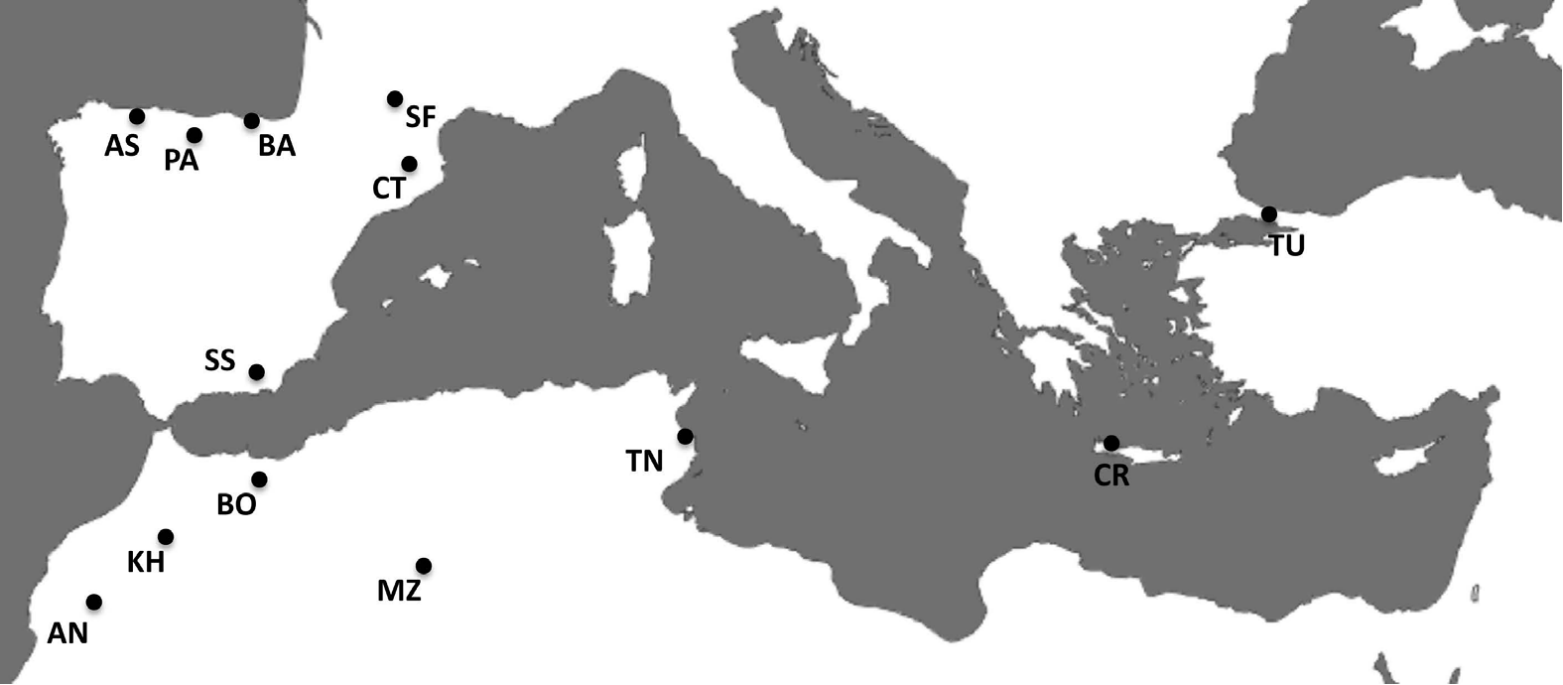
**Geographic location of the populations studied around the Mediterranean**. Sample size (in brackets) and abbreviations: AN: Asni, High Atlas, Morocco (44), AS: Oviedo, Asturias, Spain (45), BA: Basque Country, Spain (45), BO: Bouhria, Northeast Atlas, Morocco (45), CR: Crete Island, Greece (45), CT: Catalonia, Spain (45), KH: Khenifra, High Atlas, Morocco (44), MZ: M'zab, Algeria (31), PA: Pas Valley, Cantabria, Spain (44), SF: Toulouse, South France (45), SS: South Spain, Andalusia (45), TN: Monastir, Tunisia (41), TU: Istanbul, Turkey (34).

A set of 16 human populations (687 individuals) from different locations were analysed, thirteen of them originating in seven countries from around the Mediterranean: Spain (Asturias, Basque Country, Pas Valley in the north; Catalonia in the northeast; Andalusia in the south), France (Toulouse in the south), Greece (Crete island), Turkey (Istanbul), Morocco (Asni and Khenifra Berbers from High Atlas; Bouhria Berbers from Northeast Atlas), Algeria (M'zab Berbers) and Tunisia (Monastir). The location of the Mediterranean samples is shown in Figure 1. In addition, three non-Mediterranean groups (sub-Saharan Africans from the Ivory Coast; Aymaras and Quechuas from Bolivia) were included in the analysis. Sample sizes ranged from 41 to 45 individuals, with the exception of the samples from Turkey and Algeria (n = 34 and 31 respectively). Blood samples were collected for DNA extraction from healthy and unrelated individuals of both sexes and all participants had their four grandparents born in the same region. The study was performed in accordance with the guidelines of the Ethical Committee of the University of Barcelona and with informed consent of all the participants.

### Polymorphisms

**Figure 2:**
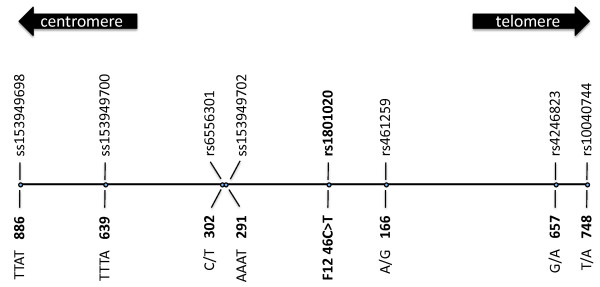
**Schematic map of the neutral and risk markers in the F12 genomic region**. The three-figure numbers in bold indicate distance from the gene (in Kbp).

In the present study, functional variation is represented by 4 SNPs and one insertion/deletion polymorphism from the F7 promoter region[12] and the 46C>T polymorphism (rs1801020) from the 5'-untranslated region in F12 exon 1,[11] also referred to as 'risk' markers. The study also included 5 more SNPs, 3 microsatellites and one SNPSTR from the wider genomic region of the F7 gene,[12] as well as 4 SNPs and 3 microsatellites from the wider genomic region of the F12 gene (Figure 2). These last 16 polymorphisms were located outside of any known genes or regulatory regions and, thus, were considered to be neutral. SNPs were selected for genotyping according to the criterion of high heterozygosity in the CEU population (US residents of northern and western European ancestry) as reported in the HapMap project http://www.hapmap.org, while microsatellites were selected as described previously[12].

### Genotype determinations

All SNPs and the insertion/deletion polymorphism were typed with the iPLEX™ Gold assay on the Sequenom MassARRAY^® ^Platform. For the microsatellites, PCR amplification was carried out, followed by 1:5 standard dilution and fragment analysis with the Applied Biosystems 3130 Genetic Analyzer[12].

### Statistical analysis

Allele frequencies of all polymorphisms were calculated with GENETIX v4.05.2[14]. Genotype frequencies were tested for goodness-of-fit to Hardy-Weinberg proportions with ARLEQUIN v3.1[15]. Additional microsatellite statistics (number of alleles; mean and variance of repeat number; and heterozygosity) were calculated using Microsatellite Analyzer (MSA) v4.05[16].

In most of the analyses that follow, our intention was to use as many of the chosen polymorphisms as possible from both the F7 and F12 genomic regions. To achieve this, we first tested all 'risk' markers for selective neutrality. Those 'risk' markers for which neutrality could not be rejected were lumped together with the neutral ones. Neutrality was tested through F_ST _comparisons: F_ST _values[17] were calculated for all loci by a locus-by-locus analysis of molecular variance (AMOVA) using ARLEQUIN. The molecular distance used was the number of pairwise differences. In the absence of selection, the F_ST _values from the 'risk' polymorphisms are expected to have the same distribution as the F_ST _values from the neutral loci. For the F7 genomic region, neutral and 'risk' F_ST _values were compared by a Mann-Whitney test[18] in R http://www.r-project.org. For the F12 genomic region, the single 'risk' 46C>T F_ST _value was compared with the 95% confidence interval from the corresponding neutral F_ST _distribution. As a final step, an additional Mann-Whitney test was carried out to check for significant differences in the patterns of variation between the markers from the two genomic regions.

In order to gain a first insight into the genetic relationships among our samples, we carried out a principal component analysis (PCA) with the *ade4 *statistical package in R,[19] using the allele frequencies of all the markers from both the F7 and F12 regions. PCA was performed for 3 different datasets: (i) all 16 populations, (ii) Old World populations only (i.e. without the Bolivian samples) and (iii) populations from the broader Mediterranean region only. PC significance was evaluated through linear correlation of PC axes with group membership of each population by an analysis of variance (ANOVA) in R. PC eigenvalues were treated as dependent variables and group membership of each population as factors. The factors used were 'South Europe', 'North Africa', 'Ivory Coast' and 'South America'.

Population structure in our samples was also surveyed by a hierarchical AMOVA (molecular distance used: number of pairwise differences) using ARLEQUIN. The input files contained the genotypic data from both the F7 and F12 genomic regions. Isolation by distance (IBD) as a possible mechanism for the observed patterns of differentiation was evaluated by a Mantel test[20] of correlation between genetic and geographic distances using the *ade4 *statistical package in R (10,000 permutations). The test was carried out for the 'Old World', 'Western Mediterranean', 'North Africa', 'South Europe' and 'Western Europe' sample subsets. The genetic distance used was that of Reynolds,[21] calculated from the allele frequencies of all the markers from both the F7 and F12 regions with PHYLIP v3.69[22]. Pairwise geographic distances (in Km) were calculated from the geographic coordinates (lat, lon) of each sample using the following formula:

To explore the possibility of a genetic boundary, we plotted together the geographic vs. genetic distances of both same-coast and opposite-coast pairs of Mediterranean samples. Genetic distances among same-coast samples similar to those among opposite-coast samples indicate that isolation by distance (IBD) is the most plausible model of differentiation. Conversely, greater genetic distances among opposite-coast samples as compared to same-coast samples would suggest that the Mediterranean is a barrier to gene flow.

We also searched for differences in gene flow from sub-Saharan Africa towards North Africa and South Europe as a potential consequence of the Mediterranean Sea acting as a genetic barrier via two different methods:

First, the degree of haplotype sharing among different groups was determined at a regional level (i.e. sub-Saharan Africa, North Africa, South Europe), as well as at a sample level (each sample treated individually); haplotypes based on both SNPs and microsatellites were inferred for each of the two genomic regions with PHASE v2.1[23,24] and were further analysed with ARLEQUIN to determine which haplotypes were shared by each pair of populations. This analysis was repeated for the SNPs alone. If the Mediterranean Sea was a genetic barrier between North Africa and South Europe, then we would expect a greater number of haplotypes to be shared between North Africans and sub-Saharans than between South Europeans and sub-Saharans, contributing also to a greater genetic differentiation between the two shores.

Second, pairwise Nm values,[25] which reflect the rate of migrant exchange between two populations, were estimated using the markers from both genomic regions with ARLEQUIN, applying the same molecular distance as in the AMOVA (see above). As ARLEQUIN returns the matrix of the *M values *(M = 2 Nm for diploid populations), we divided this matrix by a factor 2. Again, if the 'Mediterranean as a genetic barrier' hypothesis was true, we would expect higher Nm values between the Ivory Coast and each of the North African samples than between the Ivory Coast and the South Europeans. Conversely, similar numbers of shared haplotypes or Nm values would point towards the lack of a genetic barrier imposed by the Mediterranean Sea.

## Results and Discussion

### Allele frequencies, Hardy-Weinberg equilibrium and heterozygosity

After Bonferroni correction, none of the markers showed a significant departure from Hardy-Weinberg equilibrium in any population (data not shown). [Additional file 1] shows allele frequencies of the 'risk' and neutral SNPs from the F12 genomic region. The frequency of the 'risk' variant T in the polymorphism 46C>T (rs1801020) ranges from 0.081 to 0.357 in the samples from the Mediterranean countries, but was higher in the Ivory Coast (0.464) and the Native American samples (0.539 in Aymara and 0.577 in Quechua). The 46C>T frequency pattern in Native Americans was closer to that reported for Asians (C/T frequency = 0.27/0.73) than for Europeans[26]. Allele frequencies and variation statistics of the three novel microsatellites from the F12 genomic region are shown in [Additional file 2] and [Additional file 3]. Microsatellites (TTAT)_n _and (AAAT)_n _show moderate-high variability (9 alleles, total H ≈ 0.7), while tetranucleotide (TTTA)_n _is considerably less variable (6 alleles, total H = 0.181). The results from the analysis of the three microsatellites were submitted to GenBank and will become available in dbSNP Build 131. For the F7 genomic region, allele frequencies of the 'risk' and neutral biallelic, as well as microsatellite, polymorphisms were reported elsewhere[12].

### F_ST _comparisons

The F_ST _values for the 'risk' and neutral markers from the F12 genomic region are shown in Table 1. As in the case of the F7 gene,[12] the F_ST _value for the F12 46C>T 'risk' variant decreased when the Bolivian samples, first, and the Ivory Coast, second, were removed from the dataset. The 46C>T F_ST _value was not significantly different from the neutral F_ST _values in any of the three datasets considered (All Populations: 46C>T F_ST _= 0.104, neutral F_ST _95% CI [-0.012, 0.116]; Old World: 46C>T F_ST _= 0.047, neutral F_ST _95% CI [-0.015, 0.082]; Mediterranean: 46C>T F_ST _= 0.020, neutral F_ST _95% CI [-0.015, 0.050]). This last observation suggests that polymorphism 46C>T can be treated as a 'neutral' polymorphism in all the analyses of population relationships. In the F7 gene, 'risk' and neutral F_ST _values were also found not to differ significantly[12]. Finally, there seem to be no significant differences in the patterns of variation between the two genomic regions in any of the three datasets (Mann-Whitney test, p > 0.05.), which allows the fusion of the two sets of markers in all the pertinent analyses.

**Table 1:** Global F_ST _values for the neutral and risk variants (the latter in bold) of the F12 gene under 3 different datasets: All Populations; Old World samples (i.e., excluding Bolivians); and Mediterranean samples only

	All Populations	Old World	Mediterranean
(TTAT)_n_	0.028	0.025	0.004*
(TTTA)_n_	0.087	0.080	0.045
rs6556301	0.106	0.037	0.037
(AAAT)_n_	0.036	0.023	0.013
**46C>T**	**0.104**	**0.047**	**0.020**
rs461259	0.030	0.008*	0.009*
rs4246823	0.022	0.012*	0.013*
rs10040744	0.053	0.048	0.002*

Polymorphisms are listed according to their chromosomal order. An asterisk (*) indicates that the value is not significantly different from zero.

### Principal component analysis

**Figure 3:**
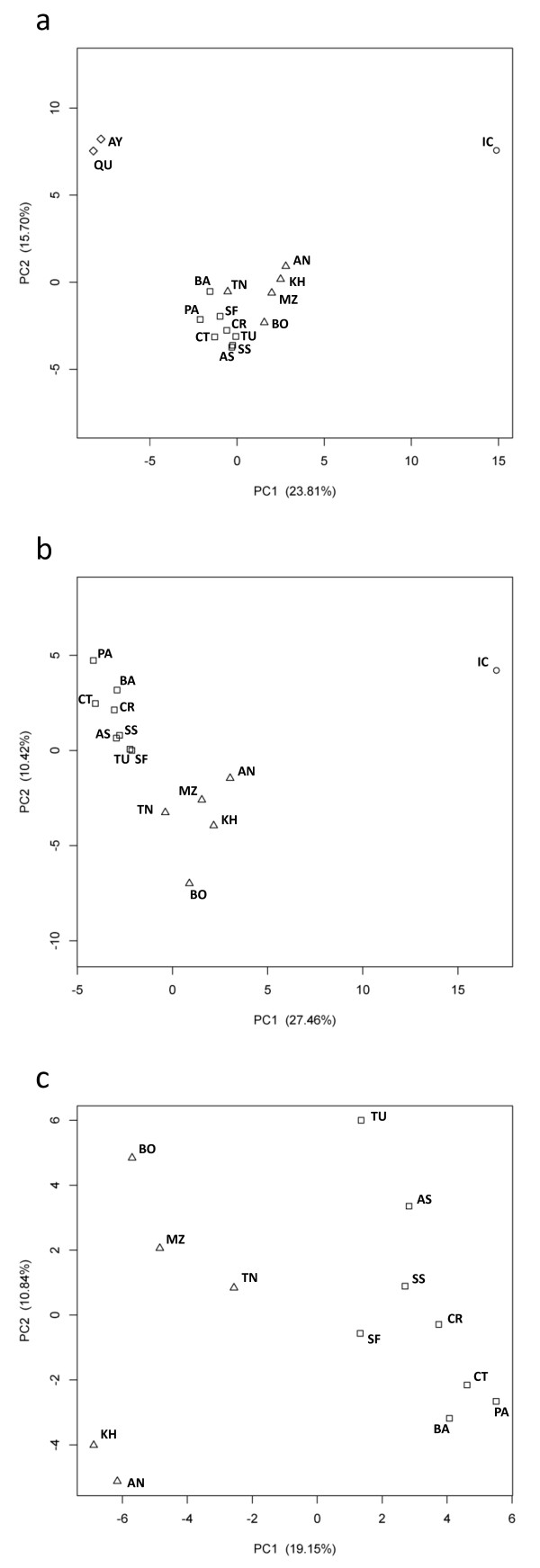
**PCA plots of the first and second PCs for: (a) All samples; (b) the Old World samples (i.e. excluding Bolivians); (c) the Mediterranean samples only**. The percentage of variation explained by each PC is shown in brackets. North African populations are represented by a triangle, South European populations by a square, Native Americans by a diamond and the Ivory Coast by a circle. PC Analyses were based on data from both the F7 and the F12 genomic regions.

Regarding population structure, when all of the populations were analysed, PCA identified three clusters, corresponding to: the Mediterranean groups; the two Bolivian groups; and the Ivory Coast (Figure 3a). In the Mediterranean cluster, all South European groups appeared separated from the North African groups along the first PC, except for Tunisia, which was closer to the South European groups. A similar result for the same Tunisian sample was reported in a previous study of Alu insertion polymorphisms on the X chromosome[27]. Although the first two PCs account for 39.52% of the original variation and the separation between North Africans and South Europeans along the second PC is visually not as clear, the population clustering according to the factors used ('South Europe', 'North Africa', 'Ivory Coast' and 'South America') was highly significant (Table 2). The ANOVA showed that the four geographical regions are clearly separated along the first PC. Populations are significantly separated along the second PC as well, although this separation is visually not as clear as in the first PC.

**Table 2:** Analysis of variance for the PC significance

	All populations		Old world populations		Mediterranean populations	
	(S.E. - N.A. - I.C. - S.AM.)		(S.E.- N.A. - I.C.)		(S.E.- N.A.)	
	
	F-quotient	p-value	F-quotient	p-value	F-quotient	p-value
PC 1	124.57	<0.01	195.68	<0.01	91.43	<0.01
PC 2	60.26	<0.01	16.22	<0.01	0.05	0.83
PC 3	15.98	<0.01	0.75	0.49	0.77	0.40
PC 4	0.09	0.97	0.35	0.71	<0.01	0.97

Abbreviations in the brackets refer to the geographical predictors used in each dataset. S.E.: South Europe, N.A.: North Africa, I.C.: Ivory Coast, S.AM.: South America.

In the PCA plot of the old world dataset (Mediterranean countries and Ivory Coast), the 13 populations from around the Mediterranean are clustered together while the Ivory Coast is positioned further away (Figure 3b). This plot revealed a clear separation between the North Africans and South Europeans along both PC1 and PC2. The North African samples are slightly closer to the sub-Saharan sample than are the European samples in PC1, but not PC2. This may reflect a higher genetic affinity of sub-Saharan Africa with North Africa than with South Europe, due to a potentially higher gene flow from the south of the Sahara towards North Africa. Clustering by population groups along the first two PCs (37.88% of the original variation) was again significant (Table 2).

In the PCA plot of just the Mediterranean dataset, the South Europe and North Africa clusters were maintained along the first PC (Figure 3c). Tunisia is the closest of the North African groups to the South Europe groups. The first two PCs account for 29.99% of the variance, although the population clustering into these two groups was significant only along the first PC (Table 2). It is also worth noting that the High Atlas Moroccans (Khenifra and Asni) seem to be separated from the other North Africans along the second PC.

### Hierarchical AMOVA

Table 3 summarises the main findings from the hierarchical AMOVA. As the decreasing values of the F-statistics indicate, the Bolivians and Ivory Coast substantially contribute to the genetic variance found in our samples: the F_ST _value decreases from 8.87% among all populations to 3.97% when the Bolivian groups are removed, to 1.67% when Ivory Coast is then removed. In the Mediterranean dataset, only 1.34% of the genetic variance was attributable to the South Europe vs. North Africa grouping (F_CT _= 0.013, p < 0.05). Comas et al.[7] and González-Pérez et al.[8] had previously studied population relationships in the western Mediterranean using autosomal Alu polymorphisms, reporting a slightly higher differentiation (F_CT _values: 0.020 and 0.018 respectively) as compared to our results. These findings suggest that the differentiation found between the two shores of the Mediterranean (also seen in all the above PC plots) is low, albeit significant.

**Table 3:** Hierarchical analysis of molecular variance based on the variation of both the F7 and the F12 genomic regions for three population datasets: All populations; Old World samples (i.e. excluding Bolivians); and Mediterranean samples only

	All	Old World	Mediterranean
Within populations	91.13	96.03	98.33
Among populations within groups	0.39	0.34	0.34
Among groups	8.52	3.64	1.34

The values correspond to the percentage of the variation explained by the corresponding grouping method. All values were statistically significant (p < 0.05).

### Genetic-geographic correlation

**Figure 4:**
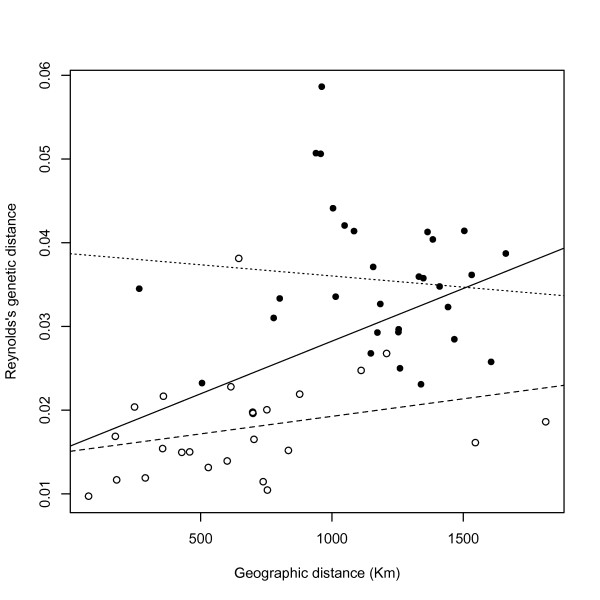
**Scatterplot of pairwise geographic vs. genetic distances of the 11 Western Mediterranean samples**. The white dots correspond to pairs of populations from the same side of the Mediterranean (i.e. NW Africa vs. NW Africa and SW Europe vs. SW Europe), while the black dots correspond to NW Africa vs. SW Europe pairs of populations. The plot reveals different distributions for the two datasets, also reflected in the respective least squares regression lines (dashed for the same-coast and dotted for the opposite-coast pairs of populations). The continuous black line represents the overall correlation.

Table 4 presents the outcome of the Mantel testing. The most significant correlation between genetic and geographic distances was observed among populations from the Western Mediterranean (i.e. Iberian Peninsula, South France and all the North Africans), suggesting that genetic differentiation in this region could be explained on the basis of just IBD. However, the plot of pairwise geographic vs. genetic distances from both same-coast and opposite-coast samples showed that, within the same range of geographic distances, opposite-coast genetic distances were greater than same-coast ones (Figure 4). This observation shows that the overall positive correlation in the Mantel test is actually driven by the larger genetic differences between populations on either side of the Western Mediterranean and the smaller genetic differences between populations on the same side. The clear separation of the two data collections in the plot seems to be consistent with the Mediterranean Sea acting as a genetic barrier, as proposed in previous studies[7].

**Table 4:** Mantel test for the significance of the correlation between genetic and geographic distance matrices for different sample subsets: Old World, western Mediterranean (i.e. without Crete and Turkey), North Africa, South Europe and Iberian Peninsula

	*r*	p	N
Old World	0.699	0.016	14
Western Mediterranean	0.478	0.005	11
North Africa	-0.083	0.531	5
South Europe	0.105	0.285	8
Western Europe	0.119	0.321	6

*r*: correlation coefficient, p: p-value, N: number of populations in each comparison.

### Haplotype sharing

Haplotype frequencies of the two genomic regions in the 3 major geographic areas (Ivory Coast, North Africa and South Europe) are shown in [Additional file 4]. For the F7 genomic region, the samples from North Africa were found to share 24 of their 225 inferred haplotypes (10.67%) with the Ivory Coast. However, an almost identical percentage (10.41%) of haplotype sharing with the Ivory Coast was found for South Europe (28 out of 269 haplotypes). When each sample was examined separately, the South European samples were found to share 6.35% - 16.33% of their inferred haplotypes (SNPs and microsatellites included) with the Ivory Coast, while the respective values for North Africans ranged between 8.33% and 18% [Additional file 5]. A Mann-Whitney test showed no significant differences between the two groups of values (data not shown). The same results were obtained when haplotypes based only on SNPs were used (data not shown). Since both shores of the Mediterranean share the same percentage of F7 haplotypes with the Ivory Coast, this genomic region does not provide any support to the hypothesis that the Mediterranean Sea obstructs sub-Saharan gene flow.

As for the F12 genomic region, 21.85% of the North African inferred haplotypes (33 out of 151) are shared with the Ivory Coast, while in South Europe this percentage falls to 13.30% (25 out of 188), suggesting higher gene flow from sub-Saharan Africa to North Africa. When each population was examined separately, the North African samples were found to share 20.34% - 28.07% of their inferred haplotypes (SNPs and microsatellites included) with the Ivory Coast, while the South European samples were found to share 12.20% - 22.22% of their inferred haplotypes with the Ivory Coast [Additional file 5]. In this case, the Mann-Whitney test showed that the above percentages in North African samples are significantly greater than those in South Europe (data not shown). However, no significant differences were found when haplotypes based only on SNPs were used (data not shown).

Although some of the above observations may indicate a higher gene flow from sub-Saharan Africa to North Africa than to South Europe, there is clearly no agreement between the two genomic regions or even between marker sets using both SNPs and microsatellites or just SNPs. Interestingly, a recent study based on autosomal Alu polymorphisms and compound Alu/microsatellite systems showed that gene flow through the Sahara was different between the two Mediterranean shores for the same sample set[28].

### Migrant exchange estimates

Migrant exchange rates for all pairs of populations as reflected by the Nm values are shown in [Additional file 6]. As seen in the first column, the range of the Nm values for each North African sample with the Ivory Coast is 2.316 - 4.887, while the same range for the South Europeans is 1.685 - 2.627. A Mann-Whitney test showed that the Nm values of the North African samples with the Ivory Coast are significantly higher than those for South Europe with the Ivory Coast (data not shown). This finding suggests that sub-Saharan gene flow, albeit of the same order of magnitude towards both sides of the Mediterranean, is higher towards North Africa, in agreement with the data based on the F12 haplotypes presented above. Since our analyses also suggest that the Mediterranean Sea acted as a barrier to gene flow between South Europe and North Africa (see Figure 4), the differences in sub-Saharan gene flow could be interpreted as a further consequence of this barrier. However, as the significant geographic-genetic correlation for the overall Old World sample set revealed, we cannot overlook that such differences in gene flow might as well be explained on the basis of geographic distance alone.

Regarding population structure in North Africa, the Nm values tended to infinity for most pairs of North African samples, indicating the lack of any barrier to migration in the region. The only exception are the High Atlas Moroccans (Asni), which show a low rate of gene flow as compared to the remaining North African samples in agreement with the extreme position of this population in the PC plots (Figure 3).

## Conclusions

With the exception of Tunisia in Figure 3a, South Europe and North Africa were always in separate clusters (Figure 3). Population structure between the two Mediterranean shores is also supported by the results of the hierarchical AMOVA, which point towards a low but significant genetic differentiation, confirming the results of previous independent studies[7,8,27]. Moreover, the F7 and F12 variation in the Western Mediterranean presents a distribution potentially compatible with the existence a genetic barrier. However, the extent to which this is true for the whole Mediterranean could not be shown here, as for such a purpose data from other important geographic regions would be necessary such as the Italian peninsula, the Adriatic Sea (e.g. Croatia and Albania) and the Northeast Africa. Finally, the data showed no consensus regarding sub-Saharan gene flow into the two sides of the Mediterranean, thereby weighing down any evaluation of its role in the North Africa vs. South Europe differentiation. The role of the Mediterranean Sea as a barrier to gene flow is still an open case.

## Authors' contributions

GA carried out the microsatellite genotyping, participated in the preparation of the DNA aliquots for SNP-typing and the statistical analyses and wrote the original manuscript. EGP carried out part of the preparation of the DNA aliquots. EE gave important advice for the improvement of the manuscript. JMD provided a substantial number of population samples to the study. MS carried out the F_ST _comparisons and language corrections and gave substantial advice for the improvement of the manuscript. PM designed and coordinated the study and participated in the draft of the manuscript. All authors read and approved the final manuscript.

## Supplementary Material

Additional file 1 **Population allele frequencies (second row for each SNP) and heterozygosities (third row and in italics) of the 5 SNPs from the F12 genomic region**. The first row for each SNP shows number of individuals typed. Polymorphisms are listed in the same order they are located on the chromosome towards the telomere. The featured frequencies correspond to the allele in bold. Click here for file

Additional file 2 **Population allele frequencies of the 3 microsatellite loci from the F12 genomic region**. Population allele frequencies of the 3 microsatellite loci from the F12 genomic region. Click here for file

Additional file 3 **Variation statistics of the 3 novel microsatellite loci from the broader F12 genomic region**. Total heterozygosity (H) refers to the heterozygosity of the pooled sample. Click here for file

Additional file 4 **Haplotype frequencies per geographic area (South Europe, North Africa, Ivory Coast) from the F7 and the F12 genomic regions**. Sheet 'F7': Haplotype frequencies per geographic area from the F7 genomic region. Microsatellite loci are separated by hyphens while SNPs and the 10 bp insertion/deletion polymorphism are not separated from each other. Numbers in microsatellite loci correspond to the repeat number. SNP and INDEL alleles are annotated by numbers '1' and '2'. The 14 polymorphisms are listed in the same order they appear on the chromosome (here seen in yellow background). Sheet 'F12': Haplotype frequencies per geographic area from the F12 genomic region. Microsatellite loci are separated by hyphens while SNPs are not separated from each other. Numbers in microsatellite loci correspond to repeat number, while SNP alleles are annotated by numbers '1' and '2'. The 8 polymorphisms are listed in the same order they appear on the chromosome (here seen in yellow background). Click here for file

Additional file 5 **Pairwise number of shared haplotypes from the F7 and F12 genomic regions**. Sheets 'F7 all markers' & 'F7 only SNPs': Pairwise number of shared haplotypes from the F7 genomic region. In sheet 'F7 all markers' estimations were based on SNPs and microsatellites, while in sheet 'F7 only SNPs' estimations were based on SNPs only. Numbers in brackets correspond to the number of distinct haplotypes found in each population. Sheets 'F12 all markers' & 'F12 only SNPs': Pairwise number of shared haplotypes from the F12 genomic region. In sheet 'F12 all markers' estimations were based on SNPs and microsatellites, while in sheet 'F12 only SNPs' estimations were based on SNPs only. Numbers in brackets correspond to the number of distinct haplotypes found in each population. Click here for file

Additional file 6 **Nm estimates per pair of Old World populations based on data from both the F7 and F12 genomic regions**. Nm estimates per pair of Old World populations based on data from both the F7 and F12 genomic regions. Click here for file
