# Supplementary material for: The Mediterranean Sea as a barrier to gene flow: evidence from variation in and around the F7 and F12 genomic regions
Source: BMC Evol Biol. 2010 Mar 27;10:84. doi: 10.1186/1471-2148-10-84 (PMC2853540; doi:10.1186/1471-2148-10-84)
Supplement: Additional file 1 — Population allele frequencies (second row for each SNP) and heterozygosities (third row and in italics) of the 5 SNPs from the F12 genomic region. The first row for each SNP shows number of individuals typed. Polymorphisms are listed in the same order they are located on the chromosome towards the telomere. The featured frequencies correspond to the allele in bold. [file 1471-2148-10-84-S1.DOC]

Additional file 1: Population allele frequencies (second row for each SNP) and heterozygosities (third row and in italics) of the 5 SNPs from the F12 genomic region

|  | **N Spain** | **NE Spain** | **Pas Valley** | **S Spain** | **Basque**  **Country** | **S France** | **Crete** | **Turkey** | **Asni Mor** | **Bouhria Mor** | **Khenifra Mor** | **M'zab Alg** | **Tunisia** | **Aymara** | **Quechua** | **Ivory Coast** |
| --- | --- | --- | --- | --- | --- | --- | --- | --- | --- | --- | --- | --- | --- | --- | --- | --- |
| rs6556301 | 43 | 45 | 42 | 45 | 43 | 43 | 44 | 31 | 40 | 41 | 41 | 28 | 40 | 42 | 41 | 38 |
| G/**T** | 0.267 | 0.333 | 0.381 | 0.300 | 0.372 | 0.349 | 0.341 | 0.323 | 0.163 | 0.110 | 0.183 | 0.107 | 0.188 | 0.655 | 0.622 | 0.171 |
|  | *0.392* | *0.444* | *0.472* | *0.420* | *0.467* | *0.454* | *0.449* | *0.437* | *0.272* | *0.195* | *0.299* | *0.191* | *0.305* | *0.452* | *0.470* | *0.284* |
| rs1801020 | 43 | 45 | 42 | 45 | 43 | 43 | 44 | 31 | 40 | 41 | 41 | 28 | 40 | 42 | 41 | 38 |
| C/**T** | 0.133 | 0.200 | 0.186 | 0.182 | 0.116 | 0.261 | 0.178 | 0.226 | 0.357 | 0.081 | 0.159 | 0.207 | 0.244 | 0.539 | 0.577 | 0.464 |
|  | *0.231* | *0.320* | *0.303* | *0.298* | *0.206* | *0.386* | *0.292* | *0.350* | *0.459* | *0.150* | *0.268* | *0.328* | *0.369* | *0.497* | *0.488* | *0.497* |
| rs461259 | 45 | 45 | 43 | 44 | 41 | 45 | 42 | 33 | 42 | 43 | 42 | 29 | 40 | 43 | 43 | 44 |
| A/**G** | 0.411 | 0.256 | 0.244 | 0.330 | 0.207 | 0.267 | 0.250 | 0.364 | 0.202 | 0.233 | 0.250 | 0.276 | 0.213 | 0.093 | 0.058 | 0.227 |
|  | *0.484* | *0.381* | *0.369* | *0.442* | *0.329* | *0.391* | *0.375* | *0.463* | *0.323* | *0.357* | *0.375* | *0.400* | *0.335* | *0.169* | *0.110* | *0.351* |
| rs4246823 | 44 | 43 | 43 | 45 | 43 | 44 | 44 | 32 | 43 | 43 | 43 | 29 | 39 | 42 | 43 | 40 |
| G/**A** | 0.318 | 0.291 | 0.372 | 0.267 | 0.384 | 0.273 | 0.239 | 0.172 | 0.291 | 0.116 | 0.302 | 0.259 | 0.282 | 0.452 | 0.419 | 0.313 |
|  | *0.434* | *0.412* | *0.467* | *0.391* | *0.473* | *0.397* | *0.363* | *0.285* | *0.412* | *0.206* | *0.422* | *0.384* | *0.405* | *0.496* | *0.487* | *0.430* |
| rs10040744 | 43 | 43 | 42 | 44 | 38 | 43 | 44 | 31 | 41 | 41 | 42 | 28 | 40 | 39 | 42 | 39 |
| T/**A** | 0.012 | 0.012 | 0.024 | 0.023 | 0.040 | 0.023 | 0.011 | 0.048 | 0.049 | 0.037 | 0.071 | 0.071 | 0.013 | 0.000 | 0.000 | 0.205 |
|  | *0.023* | *0.023* | *0.047* | *0.044* | *0.076* | *0.045* | *0.023* | *0.092* | *0.093* | *0.071* | *0.133* | *0.133* | *0.025* | *0.000* | *0.000* | *0.326* |

The first row for each SNP shows number of individuals typed. Polymorphisms are listed in the same order they are located on the chromosome towards the telomere. The featured frequencies correspond to the allele in bold.
