# Supplementary material for: The Mediterranean Sea as a barrier to gene flow: evidence from variation in and around the F7 and F12 genomic regions
Source: BMC Evol Biol. 2010 Mar 27;10:84. doi: 10.1186/1471-2148-10-84 (PMC2853540; doi:10.1186/1471-2148-10-84)
Supplement: Additional file 2 — Population allele frequencies of the 3 microsatellite loci from the F12 genomic region. Population allele frequencies of the 3 microsatellite loci from the F12 genomic region. [file 1471-2148-10-84-S2.DOC]

Additional file 2: Population allele frequencies of the 3 microsatellite loci from the F12 genomic region

|  | **N Spain** | **NE Spain** | **Pas Valley** | **S Spain** | **Basque**  **Country** | **S France** | **Crete** | **Turkey** | **Asni Mor** | **Bouhria Mor** | **Khenifra Mor** | **M'zab Alg** | **Tunisia** | **Aymara** | **Quechua** | **Ivory Coast** |
| --- | --- | --- | --- | --- | --- | --- | --- | --- | --- | --- | --- | --- | --- | --- | --- | --- |
| ss153949698 |  |  |  |  |  |  |  |  |  |  |  |  |  |  |  |  |
| (TTAT)_7_ | 0.013 |  |  | 0.011 |  |  |  | 0.030 | 0.114 | 0.019 | 0.046 | 0.083 | 0.028 |  |  | 0.197 |
| (TTAT)_8_ | 0.475 | 0.607 | 0.588 | 0.477 | 0.605 | 0.571 | 0.535 | 0.409 | 0.500 | 0.593 | 0.500 | 0.563 | 0.569 | 0.560 | 0.588 | 0.121 |
| (TTAT)_9_ | 0.100 | 0.060 | 0.118 | 0.080 | 0.105 | 0.155 | 0.151 | 0.091 | 0.102 | 0.019 | 0.046 | 0.063 | 0.028 | 0.238 | 0.221 | 0.015 |
| (TTAT)_10_ |  | 0.036 | 0.015 | 0.011 |  |  | 0.023 |  |  |  | 0.023 |  | 0.056 | 0.012 |  | 0.030 |
| (TTAT)_11_ | 0.188 | 0.179 | 0.206 | 0.216 | 0.184 | 0.131 | 0.163 | 0.288 | 0.148 | 0.204 | 0.227 | 0.063 | 0.222 | 0.179 | 0.147 | 0.182 |
| (TTAT)_12_ | 0.188 | 0.095 | 0.074 | 0.171 | 0.092 | 0.119 | 0.105 | 0.182 | 0.125 | 0.167 | 0.114 | 0.167 | 0.083 |  | 0.044 | 0.288 |
| (TTAT)_13_ | 0.038 | 0.024 |  | 0.034 |  | 0.024 | 0.023 |  | 0.011 |  | 0.046 | 0.042 |  | 0.012 |  | 0.136 |
| (TTAT)_14_ |  |  |  |  | 0.013 |  |  |  |  |  |  | 0.021 | 0.014 |  |  | 0.015 |
| (TTAT)_15_ |  |  |  |  |  |  |  |  |  |  |  |  |  |  |  | 0.015 |
|  |  |  |  |  |  |  |  |  |  |  |  |  |  |  |  |  |
| ss153949700 |  |  |  |  |  |  |  |  |  |  |  |  |  |  |  |  |
| (TTTA)_6_ |  |  |  |  |  |  |  |  | 0.012 | 0.024 | 0.026 | 0.019 | 0.013 |  |  | 0.250 |
| (TTTA)_7_ |  |  |  |  |  |  |  |  |  |  |  | 0.019 |  |  |  | 0.013 |
| (TTTA)_8_ | 1.000 | 1.000 | 0.952 | 0.947 | 0.936 | 0.954 | 0.977 | 0.936 | 0.829 | 0.744 | 0.882 | 0.833 | 0.868 | 1.000 | 0.974 | 0.650 |
| (TTTA)_9_ |  |  | 0.032 |  | 0.064 | 0.023 | 0.023 | 0.048 | 0.098 | 0.183 | 0.079 | 0.130 | 0.118 |  |  | 0.088 |
| (TTTA)_10_ |  |  |  | 0.026 |  | 0.012 |  | 0.016 | 0.024 | 0.049 |  |  |  |  |  |  |
| (TTTA)_11_ |  |  | 0.016 | 0.026 |  | 0.012 |  |  | 0.037 |  | 0.013 |  |  |  | 0.026 |  |
|  |  |  |  |  |  |  |  |  |  |  |  |  |  |  |  |  |
| ss153949702 |  |  |  |  |  |  |  |  |  |  |  |  |  |  |  |  |
| (AAAT)_6_ |  |  |  |  |  |  |  |  |  |  |  |  |  |  | 0.042 | 0.015 |
| (AAAT)_8_ |  |  |  |  | 0.038 |  |  |  |  |  | 0.046 |  | 0.029 | 0.012 | 0.014 |  |
| (AAAT)_9_ | 0.032 | 0.114 | 0.031 | 0.196 | 0.063 | 0.128 | 0.183 | 0.058 | 0.140 | 0.292 | 0.250 | 0.063 | 0.229 | 0.279 | 0.319 | 0.279 |
| (AAAT)_10_ | 0.581 | 0.398 | 0.578 | 0.411 | 0.500 | 0.423 | 0.439 | 0.462 | 0.442 | 0.486 | 0.432 | 0.604 | 0.414 | 0.616 | 0.611 | 0.279 |
| (AAAT)_11_ | 0.129 | 0.125 | 0.078 | 0.143 | 0.188 | 0.115 | 0.098 | 0.154 | 0.198 | 0.083 | 0.068 | 0.063 | 0.071 | 0.035 |  | 0.235 |
| (AAAT)_12_ | 0.242 | 0.307 | 0.156 | 0.250 | 0.150 | 0.282 | 0.232 | 0.308 | 0.209 | 0.125 | 0.182 | 0.188 | 0.257 | 0.058 |  | 0.015 |
| (AAAT)_13_ | 0.016 | 0.057 | 0.141 |  | 0.050 | 0.051 | 0.049 | 0.019 | 0.012 | 0.014 | 0.023 | 0.083 |  |  | 0.014 | 0.147 |
| (AAAT)_14_ |  |  |  |  | 0.013 |  |  |  |  |  |  |  |  |  |  | 0.029 |
| (AAAT)_15_ |  |  | 0.016 |  |  |  |  |  |  |  |  |  |  |  |  |  |
