# Supplementary material for: The Mediterranean Sea as a barrier to gene flow: evidence from variation in and around the F7 and F12 genomic regions
Source: BMC Evol Biol. 2010 Mar 27;10:84. doi: 10.1186/1471-2148-10-84 (PMC2853540; doi:10.1186/1471-2148-10-84)
Supplement: Additional file 3 — Variation statistics of the 3 novel microsatellite loci from the broader F12 genomic region. Total heterozygosity (H) refers to the heterozygosity of the pooled sample. [file 1471-2148-10-84-S3.DOC]

Additional file 3: Variation statistics of the 3 novel microsatellite loci from the broader F12 genomic region

|  | ss153949698 - (TTAT)_n_ | | | | | ss153949700 - (TTTA)_n_ | | | | | ss153949702 - (AAAT)_n_ | | | | |
| --- | --- | --- | --- | --- | --- | --- | --- | --- | --- | --- | --- | --- | --- | --- | --- |
|  | **Sample Size** | **N of Alleles** | **Mean Repeat N** | **Variance Repeat N** | **H** | **Sample Size** | **N of Alleles** | **Mean Repeat N** | **Variance Repeat N** | **H** | **Sample Size** | **N of Alleles** | **Mean Repeat N** | **Variance Repeat N** | **H** |
| N Spain | 40 | 6 | 9.59 | 3.26 | 0.701 | 19 | 1 | 8.00 | 0.00 | 0.000 | 31 | 5 | 10.63 | 0.89 | 0.596 |
| NE Spain | 42 | 6 | 9.17 | 2.60 | 0.592 | 42 | 1 | 8.00 | 0.00 | 0.000 | 44 | 5 | 10.80 | 1.36 | 0.724 |
| Pas Valley | 34 | 5 | 9.06 | 2.12 | 0.601 | 31 | 3 | 8.08 | 0.17 | 0.095 | 32 | 6 | 10.86 | 1.68 | 0.624 |
| S Spain | 44 | 7 | 9.59 | 3.16 | 0.697 | 38 | 3 | 8.13 | 0.33 | 0.102 | 28 | 4 | 10.45 | 1.16 | 0.723 |
| Basque Country | 38 | 5 | 9.11 | 2.52 | 0.588 | 39 | 2 | 8.06 | 0.06 | 0.122 | 40 | 7 | 10.55 | 1.36 | 0.693 |
| S France | 42 | 5 | 9.14 | 2.56 | 0.625 | 43 | 4 | 8.08 | 0.17 | 0.091 | 39 | 5 | 10.71 | 1.35 | 0.718 |
| Crete | 43 | 6 | 9.22 | 2.50 | 0.660 | 44 | 2 | 8.02 | 0.02 | 0.045 | 41 | 5 | 10.52 | 1.39 | 0.717 |
| Turkey | 33 | 5 | 9.65 | 2.94 | 0.718 | 31 | 3 | 8.08 | 0.11 | 0.124 | 26 | 5 | 10.77 | 1.04 | 0.678 |
| Asni Mor | 44 | 6 | 8.99 | 2.88 | 0.697 | 41 | 5 | 8.23 | 0.53 | 0.304 | 43 | 5 | 10.51 | 1.03 | 0.711 |
| Bourhia Mor | 27 | 5 | 9.28 | 2.96 | 0.590 | 41 | 4 | 8.23 | 0.43 | 0.415 | 36 | 5 | 10.08 | 1.01 | 0.665 |
| Khenifra Mor | 44 | 7 | 9.41 | 3.23 | 0.687 | 38 | 4 | 8.07 | 0.30 | 0.219 | 22 | 6 | 10.16 | 1.44 | 0.727 |
| M'zab Alg | 24 | 7 | 9.17 | 3.89 | 0.652 | 27 | 4 | 8.07 | 0.22 | 0.294 | 24 | 5 | 10.63 | 1.26 | 0.598 |
| Tunisia | 36 | 7 | 9.19 | 2.72 | 0.623 | 38 | 3 | 8.09 | 0.16 | 0.235 | 35 | 5 | 10.30 | 1.37 | 0.714 |
| Aymara | 42 | 5 | 8.86 | 1.47 | 0.605 | 43 | 1 | 8.00 | 0.00 | 0.000 | 43 | 5 | 9.85 | 0.58 | 0.544 |
| Quechua | 34 | 4 | 8.84 | 1.57 | 0.590 | 39 | 2 | 8.08 | 0.23 | 0.051 | 36 | 5 | 9.53 | 0.96 | 0.530 |
| Ivory Coast | 33 | 9 | 10.45 | 5.33 | 0.823 | 40 | 4 | 7.58 | 0.93 | 0.514 | 34 | 7 | 10.49 | 2.40 | 0.777 |
| Total | 600 | 9 | 9.29 | 2.92 | 0.672 | 594 | 6 | 8.05 | 0.26 | 0.181 | 554 | 9 | 10.42 | 1.37 | 0.695 |

Total heterozygosity (H) refers to the heterozygosity of the pooled sample.
