# Supplementary material for: The Mediterranean Sea as a barrier to gene flow: evidence from variation in and around the F7 and F12 genomic regions
Source: BMC Evol Biol. 2010 Mar 27;10:84. doi: 10.1186/1471-2148-10-84 (PMC2853540; doi:10.1186/1471-2148-10-84)
Supplement: Additional file 6 — Nm estimates per pair of Old World populations based on data from both the F7 and F12 genomic regions. Nm estimates per pair of Old World populations based on data from both the F7 and F12 genomic regions. [file 1471-2148-10-84-S6.DOC]

Additional file 6: Nm estimates per pair of Old World populations based on data from both the F7 and F12 genomic regions

|  | Ivory Coast | South Spain | Asni Morocco | North Spain | Bouhria Morocco | Crete | Khenifra Morocco | M’zab Algeria | Northeast Spain | Pas Valley | Tunisia | South France | Turkey |
| --- | --- | --- | --- | --- | --- | --- | --- | --- | --- | --- | --- | --- | --- |
| South Spain | 2.110 |  |  |  |  |  |  |  |  |  |  |  |  |
| Asni Morocco | 4.887 | 20.923 |  |  |  |  |  |  |  |  |  |  |  |
| North Spain | 2.422 | ∞ | ∞ |  |  |  |  |  |  |  |  |  |  |
| Bouhria Morocco | 2.316 | 134.377 | 62.281 | ∞ |  |  |  |  |  |  |  |  |  |
| Crete | 2.056 | ∞ | 14.454 | ∞ | 15.659 |  |  |  |  |  |  |  |  |
| Khenifra Morocco | 4.133 | 30.257 | ∞ | ∞ | ∞ | ∞ |  |  |  |  |  |  |  |
| M’zab Algeria | 2.828 | 18.336 | 150.487 | ∞ | ∞ | 15.008 | ∞ |  |  |  |  |  |  |
| Northeast Spain | 1.685 | ∞ | 10.408 | ∞ | 9.764 | ∞ | 330.051 | 10.143 |  |  |  |  |  |
| Pas Valley | 1.969 | ∞ | 16.433 | ∞ | 8.059 | ∞ | 23.478 | 9.600 | ∞ |  |  |  |  |
| Tunisia | 2.943 | 40.764 | 69.542 | ∞ | ∞ | 23.667 | ∞ | ∞ | 13.329 | 13.786 |  |  |  |
| South France | 2.104 | ∞ | 25.789 | ∞ | 13.233 | 700.014 | ∞ | 22.795 | ∞ | ∞ | 21.754 |  |  |
| Turkey | 2.627 | ∞ | 38.167 | ∞ | 22.995 | ∞ | ∞ | 58.526 | 665.564 | 251.752 | 171.146 | ∞ |  |
| Basque Country | 1.830 | ∞ | 16.290 | ∞ | 7.217 | 106.837 | ∞ | 13.234 | 6334.273 | ∞ | 27.058 | 81.579 | 27.872 |
